# Narrative nursing for prolonged myelosuppression after autologous stem cell transplantation in multiple myeloma: A case report

**DOI:** 10.1097/MD.0000000000051028

**Published:** 2026-10-02

**Authors:** Qionglei Huang, Min Jiang

**Affiliations:** a Department of Nursing, Clinical College of Anhui Medical University, Hefei, Anhui Province, China.

**Keywords:** autologous stem cell transplantation, case report, multiple myeloma, myelosuppression, narrative nursing

## Abstract

**Rationale:**

Myelosuppression after autologous stem cell transplantation (ASCT) exposes patients with multiple myeloma to substantial infection and bleeding risks and may be accompanied by marked psychological distress during protective isolation. Although supportive care is central to posttransplant recovery, reports describing how narrative nursing can be integrated into routine transplantation care remain limited.

**Patient concerns:**

A 46-year-old married man with multiple myeloma was admitted 7 months after ASCT because of fever for half a day and erosive oral-labial mucosal lesions. He presented with febrile neutropenia, oral mucositis, severe pancytopenia, anxiety, hopelessness, and concern about his family role as the main financial provider.

**Diagnoses:**

The patient was diagnosed with multiple myeloma after ASCT complicated by prolonged myelosuppression, febrile neutropenia, oral mucositis, and severe pancytopenia. Delayed hematopoietic reconstitution and infection, including possible fungal infection, were considered during the clinical course.

**Interventions:**

The patient received protective isolation in a laminar airflow room, empiric broad-spectrum antimicrobial therapy, granulocyte colony-stimulating factor, hemostatic prophylaxis, blood component support, meticulous oral and skin-mucosal care, nutrition and hydration support, and dynamic monitoring. A supplemental infusion of autologous hematopoietic stem cells was administered on hospital day 2. Individualized narrative nursing was provided through repeated one-to-one conversations, emotional validation, misconception correction, achievable daily goal setting, family communication, and discharge planning.

**Outcomes:**

During the 16-day hospitalization, fever and mucositis resolved, leukocyte and neutrophil counts gradually recovered, inflammatory markers declined, and no new infection or major bleeding occurred. The patient reported reduced fear, improved sleep, and greater willingness to participate in care and follow-up.

**Lessons:**

This case suggests that structured narrative nursing can be feasibly embedded in routine ASCT supportive care during prolonged myelosuppression and may support emotional adjustment, treatment engagement, and family-centered coping.

## 1. Introduction

Multiple myeloma (MM) is a plasma cell malignancy characterized by clonal plasma cell proliferation and monoclonal immunoglobulin production, leading to organ damage such as anemia, bone disease, renal impairment, infection, and hypercalcemia.^[1,2]^ Autologous stem cell transplantation (ASCT) remains a key component of therapy for eligible patients and can improve survival and quality of life.^[3]^ Conditioning regimens and delayed hematopoietic recovery may cause profound myelosuppression, increasing the risk of febrile neutropenia, mucositis, bleeding, and other complications that require comprehensive supportive care.^[4,5]^ In addition to physical burden, patients undergoing hematopoietic stem cell transplantation frequently experience clinically significant anxiety and depressive symptoms during hospitalization and isolation, which may adversely affect symptom burden and treatment adherence.^[6]^

Narrative nursing, informed by principles of narrative medicine, emphasizes attentive listening, empathic reflection, and meaning-making to help patients articulate and reframe illness experiences.^[7]^ Evidence from transplantation-related supportive care and psychosocial interventions suggests that structured communication may improve quality of life, coping, and emotional well-being.^[8,9]^ Here, we report the diagnosis, clinical course, supportive treatment, and narrative nursing care of a patient with MM who developed prolonged myelosuppression and infection risk after ASCT, highlighting practical nursing priorities during protective isolation and recovery. This case report was prepared in accordance with the CARE reporting guideline.

## 2. Case presentation

### 2.1. General information

A 46-year-old married man with a high school education was admitted on August 8, 2024 because of fever for half a day and erosive lesions of the oral-labial mucosa. He had been diagnosed with MM 12 months earlier and had undergone ASCT 7 months before this admission. Hematopoietic recovery after ASCT had been poor, requiring intermittent blood product support. The patient was the main financial provider for his family, which contributed to significant role-related stress during hospitalization. Ethical approval for this case report was waived by the Clinical College of Anhui Medical University because this was a retrospective report of a single patient without any deviation from standard clinical care. Written informed consent for publication of the clinical details was obtained from the patient.

### 2.2. Clinical findings and diagnostic assessment

On admission, body temperature was 38.7°C, pulse was 104 beats/min, respiratory rate was 22 breaths/min, and blood pressure was 110/70 mm Hg. Physical examination showed crusted erosions on the lips and mild pharyngeal hyperemia. Breath sounds were slightly coarse without focal findings. Laboratory tests showed severe pancytopenia, with a white blood cell count of 0.21 × 10^9^/L, hemoglobin of 55 g/L, and platelet count of 12 × 10^9^/L. Inflammatory markers were elevated, consistent with a systemic inflammatory response.

The patient was assessed as having MM after ASCT complicated by prolonged myelosuppression, febrile neutropenia, oral mucositis, and severe pancytopenia. During hospitalization, the absolute neutrophil count was <0.1 × 10^9^/L, indicating profound neutropenia. The duration of cytopenia exceeded the expected recovery window after standard high-dose melphalan conditioning, raising concern for delayed hematopoietic reconstitution. Diagnostic reasoning also considered bacterial infection, possible fungal infection, disease- or treatment-related marrow suppression, and impaired stem cell reserve. Bone marrow suppression following ASCT is typically characterized by transient aplasia lasting 7 to 14 days; however, delayed engraftment may occur in association with prior intensive chemotherapy exposure, infection, nutritional compromise, or impaired stem cell reserve.

### 2.3. Therapeutic interventions and nursing process

The patient was placed in a laminar airflow room with protective isolation. Empiric intravenous antibiotics were initiated, granulocyte colony-stimulating factor was administered, hemostatic prophylaxis was provided, and blood components were transfused according to hemoglobin and platelet levels. Supportive care included oral and skin-mucosal care, nutrition and hydration support, and dynamic monitoring of vital signs, fluid balance, blood counts, and inflammatory indices. Given persistently poor hematopoietic recovery and high infection risk, a supplemental infusion of autologous hematopoietic stem cells (140 mL) was administered on hospital day 2 without infusion-related adverse reactions. Because of the prolonged disease course and concern for fungal infection, voriconazole was added.

Persistent fever, prolonged cytopenia, and separation from family in protective isolation aggravated anxiety and hopelessness. The patient stated, “I’ve been sick for so long and I’m still not getting better,” and expressed concern about being the main financial provider for his family. The primary nurse delivered repeated narrative nursing sessions to elicit the illness story, validate emotions, clarify misconceptions, reframe myelosuppression as an expected and time-limited phase, and support goal setting, including airway clearance practice and sleep improvement. Family members were coached to provide supportive language and participate in a shared coping narrative. The timeline of the clinical course and narrative nursing interventions is summarized in Table 1.

**Table 1:** Clinical course and narrative nursing interventions.

Day	Clinical status	Supportive care bundle	Narrative nursing focus
0	Fever, mucositis, severe pancytopenia	Isolation; monitoring; empiric intravenous antibiotics; G-CSF; transfusion as needed; oral and skin care	Invite illness story; validate fear and role strain; assess coping
2	Supplemental autologous stem cell infusion	Aseptic infusion care; pre-infusion education; close vital signs monitoring; adverse reaction observation	Reframe myelosuppression as expected and time-limited; set 1–2 daily achievable goals
7–10	Symptoms improving; counts rising	Continue infection and bleeding precautions; nutrition and hydration support; airway clearance coaching	Identify turning points; reinforce self-efficacy; strengthen coping narrative
13	Afebrile or low-grade temperature; inflammation declining	Adjust antimicrobials as needed; sleep hygiene; discharge preparation	Encourage brief reflection recording of progress; guide family supportive language
16	Stable condition; ready for discharge	Self-care instructions; warning signs; follow-up plan; medication guidance	Consolidate recovery story; plan follow-up contact

G-CSF = granulocyte colony-stimulating factor.

### 2.4. Outcomes and follow-up

Around hospital days 7 to 10, temperature fluctuations decreased, cough and mucositis improved, and leukocyte and neutrophil counts began to rise gradually. From hospital day 13, body temperature remained below 37.3°C, inflammatory markers declined, and hemoglobin and platelet levels improved. The patient reported, “I’m not as scared as when I first came.”

On hospital day 16, fever had resolved, oral lesions had healed, no new infection or major bleeding had occurred, and blood counts were close to recovery thresholds for discharge. Mood and sleep improved, and the patient expressed willingness to adhere to follow-up and ongoing therapy. Discharge education emphasized infection warning signs, bleeding precautions, oral care, medication adherence, nutrition, follow-up review, and continuation of brief narrative reflection, such as recording small improvements and turning points during recovery.

## 3. Nursing

### 3.1. Protective isolation and infection/bleeding prevention

During profound neutropenia, nurses implemented protective isolation, strict hand hygiene, environmental disinfection, visitor management, and close monitoring of temperature, respiratory symptoms, oral lesions, skin condition, catheter sites, and laboratory indicators. Bleeding precautions were reinforced because of severe thrombocytopenia, including careful oral care, avoidance of trauma, monitoring for skin ecchymosis and mucosal bleeding, and timely reporting of abnormal bleeding signs. These measures supported early recognition of infection or bleeding and helped guide timely medical adjustment.

### 3.2. Stem cell infusion and hematologic supportive care

For the supplemental autologous stem cell infusion, nursing care focused on pre-infusion education, verification of patient identity and infusion details, aseptic technique, close monitoring of vital signs, and prompt recognition of infusion-related reactions. The infusion was completed without adverse reactions. Nurses also coordinated transfusion-related observation, monitored hemoglobin and platelet trends, and supported administration of granulocyte colony-stimulating factor and other prescribed therapies.

### 3.3. Oral mucosal, skin, nutritional, and symptom care

Oral mucositis and lip erosions were managed through meticulous oral hygiene, mucosal assessment, symptom observation, and education on gentle oral care. Skin and mucosal integrity were assessed regularly to reduce infection entry points. Nutritional and hydration support were provided according to tolerance, and nurses encouraged adequate intake when symptoms improved. Airway clearance coaching, rest planning, and sleep hygiene were incorporated into daily care goals to improve comfort and participation.

### 3.4. Narrative nursing and family-centered continuing care

Narrative nursing was integrated into routine care through a repeated cycle of telling, listening, empathic response, and reframing. During daily rounds, before or after procedures, and at quiet times in the evening, the primary nurse initiated nondirective conversations and used open-ended prompts, such as “What has been the hardest part recently?” and “What worries you most about the transplant?” Active listening, silence, reflection, and summarizing helped reduce the patient’s sense of isolation.

Meaning reconstruction was co-developed with the patient. Misconceptions such as “myelosuppression means the disease is worsening” were gently clarified and reframed as an expected, time-limited “tunnel period” after high-dose therapy. Concrete micro-goals, including effective nebulization and sputum clearance, incremental sleep extension, and completion of oral care, were negotiated and reviewed daily. Family members were coached to use validating and collaborative language, such as “we will work through this step by step,” and to avoid blame or catastrophic framing. After discharge, the patient was encouraged to continue brief diaries, voice notes, or short videos recording small wins and turning points, which could be revisited during follow-up.

## 4. Discussion

Delayed hematopoietic recovery after ASCT remains a clinically relevant complication. Although high-dose melphalan conditioning predictably induces marrow aplasia, successful engraftment depends on adequate stem cell dose, microenvironmental support, absence of severe infection, and preserved host immune regulation. Prolonged cytopenia has been associated with infectious morbidity, extended hospitalization, and higher care burden. Risk factors reported in the literature include older age, prior cumulative chemotherapy exposure, suboptimal CD34+ cell dose, infection, nutritional compromise, and inflammatory dysregulation. Therefore, early identification of delayed recovery and prompt institution of growth factor support, transfusion management, and infection prevention are essential components of posttransplant care.^[3–5]^

Comprehensive supportive care is essential during the myelosuppression period after ASCT, including strict infection prevention, bleeding risk management, nutrition support, and meticulous mucosal and skin care.^[4,5]^ Standardized nursing for stem cell infusion, including pre-infusion education, aseptic technique, close monitoring of vital signs, and prompt recognition and management of adverse events, helps ensure procedural safety.^[3]^ In this case, protective isolation, antimicrobial therapy, hematologic support, stem cell infusion care, and meticulous mucosal care provided the biological foundation for recovery.

Psychological distress is common during transplantation hospitalization and protective isolation. Anxiety and depressive symptoms may undermine adherence, sleep, self-care, and recovery.^[6]^ Beyond behavioral pathways, distress may also have biological relevance. Higher pretransplant anxiety and depression have been associated with slower early leukocyte recovery after autologous hematopoietic cell transplantation, suggesting a potential link between affective symptoms and immune reconstitution.^[10]^ In this case, prolonged cytopenia, uncertainty about recovery, isolation from family, and role strain as the family’s financial provider amplified fear, helplessness, and loneliness.

Narrative nursing provided a practical, patient-centered framework to address these concerns by strengthening meaning-making and agency. Narrative medicine highlights clinicians’ narrative competence, including attention, reflection, and affiliation, as foundations for empathic partnership and trust.^[7]^ In nursing, story theory conceptualizes a narrative happening that unfolds through intentional nurse-person dialogue, supporting reconstruction of the illness story and movement toward resolving the complicating health challenge.^[11]^ Guided by these principles, the team integrated narrative nursing into routine care through repeated conversations, emotional validation, cognitive reframing, achievable goal setting, and family communication.

Family-centered narrative work may be particularly valuable in transplantation care. Family functioning is clinically relevant in hematopoietic stem cell transplantation trajectories; higher family cohesion has been associated with lower symptom distress over time, supporting the value of family-centered communication strategies.^[12]^ Storytelling-based psychosocial interventions in hematopoietic cell transplantation patient-caregiver dyads also support the feasibility of using shared narratives to strengthen coping.^[9]^ In this case, family members were encouraged to provide validating language and participate in a shared recovery narrative, which may have helped the patient feel less isolated and more engaged in care.

From a transplant medicine perspective, integrating psychosocial support within biologically driven supportive management may offer additive benefits. Hematologic recovery is primarily determined by stem cell kinetics and marrow regeneration; however, patient engagement, adherence to infection prevention protocols, nutritional intake, sleep, and early symptom reporting are influenced by psychological status. Thus, structured communication strategies may indirectly support physiologic recovery by promoting behavioral adherence and reducing stress-related vulnerability.

## 5. Conclusion

This case highlights that patients with MM experiencing prolonged myelosuppression after ASCT require integrated supportive care that addresses infection risk, bleeding risk, mucosal injury, hematologic recovery, psychological distress, and family coping. Structured narrative nursing can be embedded into routine transplantation nursing care and may support emotional adjustment, treatment engagement, adherence, and family-centered recovery during protective isolation and discharge transition.

## 6. Limitations

This report describes a single patient; therefore, the findings may not be generalizable. Standardized psychological measures were not collected longitudinally, and detailed time-stamped laboratory trajectories were limited. Additional details such as CD34+ cell dose, complete antimicrobial dosing, and MM staging were unavailable in the current manuscript records. Future studies should combine patient-reported outcomes, structured qualitative narrative data, and serial hematologic indices to improve replicability and evaluate the mechanisms and effectiveness of narrative nursing in ASCT settings.

## 7. Patient perspective

The patient reported that repeated narrative conversations helped him feel understood and less isolated during protective isolation. He stated that he was “not as scared as when I first came” and became more willing to participate in oral care, airway clearance, and follow-up planning. He also expressed that setting small daily goals helped him notice progress and regain confidence during a period of uncertainty.

## Acknowledgments

The authors thank the patient for consenting to the publication of this case report. This work was supported by the Natural Science Research Project of Universities in Anhui Province, 2023 (No. 2023AH052768). The authors declare no conflicts of interest.

## Author contributions

**Writing – original draft:** Min Jiang.

**Writing – review & editing:** Qionglei Huang.
